# Mutilating localized cutaneous leishmaniasis caused by *Leishmania guyanensis*^☆^

**DOI:** 10.1016/j.abd.2022.04.014

**Published:** 2023-02-28

**Authors:** Dina Fabrício da Silva, Sidharta Quercia Gadelha, Andréa de Souza Cavalcante, Rosilene Viana de Andrade, Jorge Augusto de Oliveira Guerra, Alcidarta dos Reis Gadelha

**Affiliations:** a Department of Dermatology, Fundação de Dermatologia Tropical Heitor Vieira Dourado, Manaus, AM, Brazil; b Department of Leishmaniasis, Fundação de Dermatologia Tropical Heitor Vieira Dourado, Manaus, AM, Brazil; c Department of Dermatopathology, Fundação de Dermatologia Tropical Heitor Vieira Dourado, Manaus, AM, Brazil

Dear Editor,

American tegumentary leishmaniasis (ATL) is an infectious disease caused by protozoa of the genus *Leishmania* transmitted by the bite of a female sandfly of the genus *Lutzomyia*.^1^ In Brazil, it is found in all states, with Amazonas and Pará accounting for 58% of reported cases between 2007 and 2017.^2^ The three main species responsible for ATL in Brazil are *L.(V.) braziliensis*, *L.(V.) guyanensis*, and *L.(L.) amazonensis*.^1^ Epidemiological data and polymerase chain reaction (PCR) analysis may be essential for the diagnosis of unusual clinical forms, and/or in the absence of amastigotes in smears and histological sections.

## Case report

**Figure 1:**
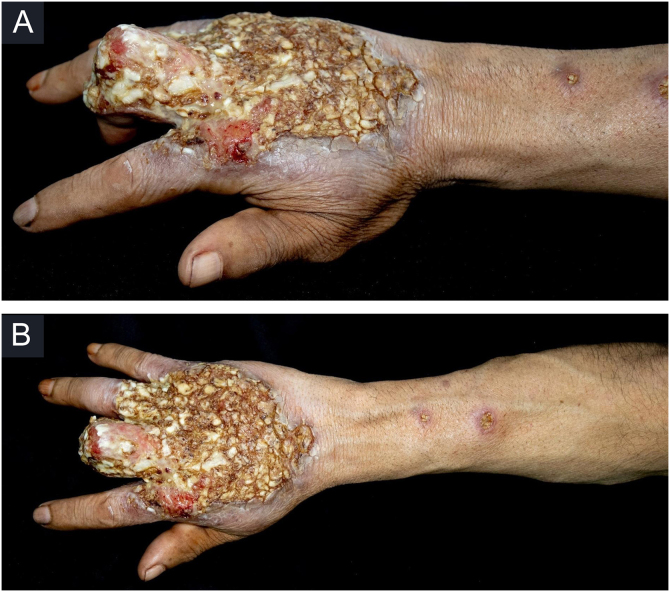
(A) Ulcer on the dorsum of the right hand covered by yellowish crusts, with clear erythematous borders extending to the phalanges with anatomical deformity of the third finger. (B) Ulcerated nodules following an ascending lymphatic path in the right forearm.

**Figure 2:**
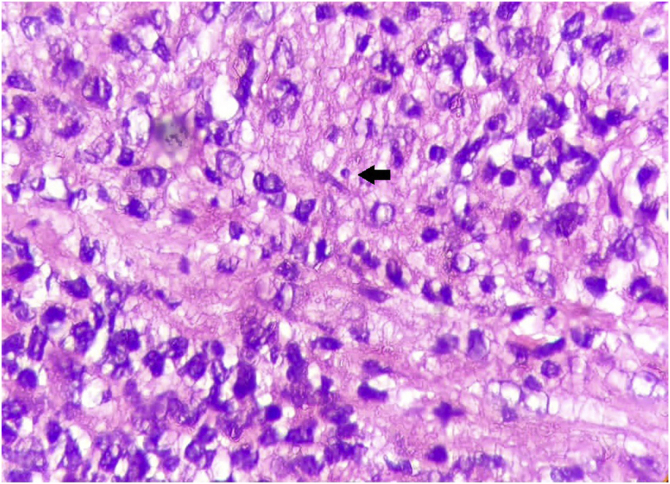
Histopathological examination. Hematoxylin & eosin, ×1000. Granulomatous inflammation consisting of plasmocytes, lymphocytes, histiocytes, epithelioid cells, and the presence of rare amastigotes (arrow).

**Figure 3:**
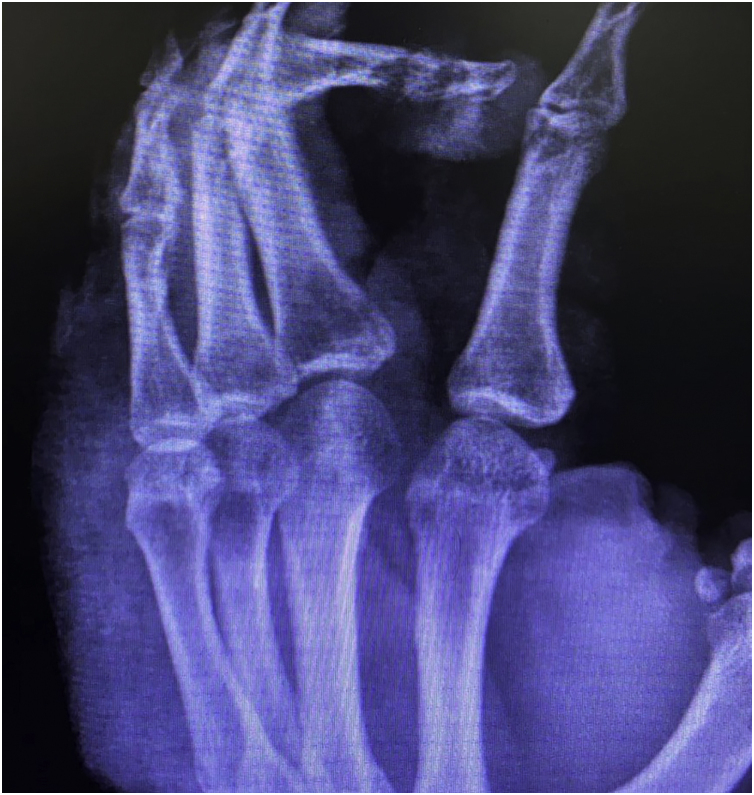
Right-hand x-ray. Presence of bone sequestration, and destruction of the cortex of the distal and middle phalanges of the third finger, suggestive of chronic osteomyelitis.

**Figure 4:**
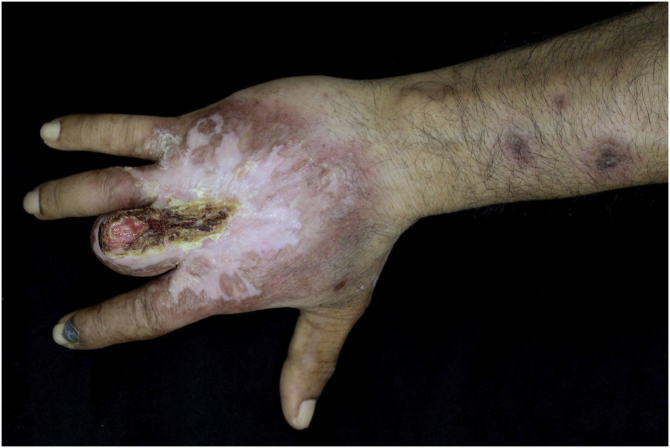
Significant improvement and regression of the crusted ulcer after treatment with meglumine antimoniate for 30 days.

This case report describes a 49-year-old male patient, a farmer, without comorbidities, from Maués, state of Amazonas, Brazil. He sought medical care at a Dermatology service complaining of a condition which had started three years before, with a papule on the proximal phalanx of the third finger, progressing to an ulcer that extended to the dorsum of the right hand. Considering the clinical picture, nine direct tests were performed to search for amastigotes, all with negative results. Different types of treatments with antibiotics and corticoids were prescribed without improvement, and with worsening and progression of the lesions for the last six months. On examination, an ulcer was observed on the dorsum of the right hand, covered by yellowish crusts, with clear and erythematous borders, which extended to the phalanges, resulting in anatomical deformity of the third finger. There were also nodules following an ascending lymphatic path, in the right forearm, some of which showed painless ulceration, with raised erythematous borders, measuring approximately 2 cm (Figs. 1A and 1 B). Histopathological examination of biopsies from the lesion located on the dorsum of the hand and from a nodule on the forearm revealed the presence of granulomatous inflammatory infiltrate consisting of histiocytes, epithelioid cells, giant cells, several plasmocytes, lymphocytes, and the presence of rare amastigotes (Fig. 2). The PCR sequencing, carried out with the aid of primers described by Graça et al.,^3^ on the biopsy fragment identified the species *Leishmania guyanensis*. The bacteriological culture of the fragment from the dorsum of the hand was positive for the Gram-negative bacillus *Pseudomonas aeruginosa*. The radiological examination showed bone sequestration and destruction of the cortex of the distal and middle phalanges of the third finger, suggestive of chronic osteomyelitis (Fig. 3). The combined treatment consisting of meglumine antimoniate 1200 mg of Sb^+5^/day intravenously for 30 days and cefepime, according to the sensitivity identified in the antibiogram, at a dose of 1 g 2×/day for 10 days, resulted in significant improvement in the skin lesions (Fig. 4). The patient is currently undergoing multidisciplinary follow-up with the orthopedics service for surgical treatment of the osteomyelitis after regression of the skin ulcer.

## Discussion

ATL can be classified into localized cutaneous, disseminated, diffuse, and mucocutaneous leishmaniasis.^1^ The typical lesion of localized cutaneous leishmaniasis is initially a papule that progresses to an ulcer with a raised, infiltrated border and a granular background, which may be accompanied by regional lymphadenopathy and/or nodular lymphangitis.^1^ However, the clinical forms of pleomorphic and atypical aspects can hinder and delay the diagnosis and, consequently, adequate treatment.^4^

Among the atypical forms of ATL, the verrucous, lupoid, eczematous, zosteriform, tumor, acneiform, and sporotrichoid forms have been reported.^5^, ^6^ Pazok Hossein et al. described cases similar to the one described in the present case report as lupoid variants of ATL in patients from Afghanistan and Turkey, respectively.^7^

The diagnosis should be based on epidemiology, lesion characteristics, direct examination, and culture. In atypical cases, histopathological examination and PCR are fundamental tools.^1^, ^5^

Direct examination is the most frequently used method for diagnosis because it is simple, inexpensive and easy to perform. However, the probability of finding the parasite is inversely proportional to the time of evolution of the skin lesions, being rare after one year.^1^

On histopathology, intense granulomatous inflammatory infiltrate is observed, and histiocytes, epithelioid and giant cells, lymphocytes, plasma cells, some eosinophils, and depending on the time of evolution, macrophages containing amastigotes can be seen.^5^

PCR, a method based on parasite DNA amplification, has a sensitivity and specificity of 100% in the typical forms and a sensitivity of 94% in atypical presentations.^1^, ^5^ In this case, PCR compatible with *Leishmania guyanensis* was crucial for the correct diagnosis and starting treatment.

The rare bone involvement reported in cases of ATL and proven infection by *Pseudomonas aeruginosa*, as well as the radiological alterations suggestive of persistent chronic osteomyelitis in the radiological control, suggest that the bone lesions observed in the present case were caused by contiguity from the secondary infection.^8^ However, histopathological examination and culture of the bone lesion are necessary.

Despite its wide distribution in Brazil, atypical presentations of ATL can make the diagnosis difficult. Therefore, the relevance of epidemiology and PCR in atypical lesions is highlighted, as well as the importance of an early diagnosis, so that sequelae or mutilations can be prevented.

## Financial support

None declared.

## Authors' contributions

Dina Fabrício da Silva: Approval of the final version of the manuscript; drafting and editing of the manuscript; collection, analysis, and interpretation of data; intellectual participation in the propaedeutic and/or therapeutic conduct of the case; critical review of the literature; critical review of the manuscript.

Sidharta Quercia Gadelha: Approval of the final version of the manuscript; drafting and editing of the manuscript; collection, analysis, and interpretation of data; intellectual participation in the propaedeutic and/or therapeutic conduct of the case; critical review of the literature; critical review of the manuscript.

Andréa de Souza Cavalcante: Approval of the final version of the manuscript; design and planning of the study; intellectual participation in the propaedeutic and/or therapeutic conduct of the case; critical review of the literature; critical review of the manuscript.

Rosilene Viana de Andrade: Approval of the final version of the manuscript; design and planning of the study; participation in the writing of the histopathological report; critical review of the literature; critical review of the manuscript.

Jorge Augusto de Oliveira Guerra: Approval of the final version of the manuscript; design and planning of the study; intellectual participation in the propaedeutic and/or therapeutic conduct of the case; critical review of the literature; critical review of the manuscript.

Alcidarta dos Reis Gadelha: Approval of the final version of the manuscript; design and planning of the study; intellectual participation in the propaedeutic and/or therapeutic conduct of the case; critical review of the literature; critical review of the manuscript.

## Conflicts of interest

None declared.
